# Non-Newtonian Flow to the Theoretical Strength of Glasses via Impact Nanoindentation at Room Temperature

**DOI:** 10.1038/s41598-017-17871-4

**Published:** 2017-12-15

**Authors:** Christoffer Zehnder, Jan-Niklas Peltzer, James S. K.-L. Gibson, Doris Möncke, Sandra Korte-Kerzel

**Affiliations:** 1 Institute of Physical Metallurgy and Metal Physics, RWTH Aachen University, Aachen, Germany; 2 Department of Built Environment and Energy Technology, Linnaeus University, Växjö, Sweden; 3 Theoretical and Physical Chemistry Institute, National Hellenic Research Foundation, Athens, Greece

## Abstract

In many daily applications glasses are indispensable and novel applications demanding improved strength and crack resistance are appearing continuously. Up to now, the fundamental mechanical processes in glasses subjected to high strain rates at room temperature are largely unknown and thus guidelines for one of the major failure conditions of glass components are non-existent. Here, we elucidate this important regime for the first time using glasses ranging from a dense metallic glass to open fused silica by impact as well as quasi-static nanoindentation. We show that towards high strain rates, shear deformation becomes the dominant mechanism in all glasses accompanied by Non-Newtonian behaviour evident in a drop of viscosity with increasing rate covering eight orders of magnitude. All glasses converge to the same limit stress determined by the theoretical hardness, thus giving the first experimental and quantitative evidence that Non-Newtonian shear flow occurs at the theoretical strength at room temperature.

## Introduction

Glass shapes our way of life; from the basic needs of shelter and light to the modern ever-presence of the global networks on mobile devices. Yet deformation of glasses in exactly these applications, from static windows to flexibly loaded and frequently damaged displays, is in many aspects only poorly understood. Our current understanding is focused on the catastrophic fracture events in glasses, not their plastic deformation. In metals, it is the physical understanding of how deformation takes place down to the atomic level which has enabled scientists to improve technical alloys from steels to superalloys at staggering rates over the last decades. Unlike metals, glasses exhibit a short range order (SRO) represented by a few atoms building recurring structural units, but these do not stack periodically in three dimensions to give long range order (LRO). The underlying mechanisms in glasses must be inherently different compared to metals, due to the lack of crystalline order. Nevertheless, it is plastic deformation, either by densification of the glass network or shear flow, which accommodates scratches on transparent devices and, more importantly, competes with or pre-mediates fracture. To investigate and understand these mechanisms is crucial where new technologies, with their rapidly growing demands on strength and toughness of the materials and composites made from them, are to be implemented efficiently.

Until now, a vast yet most relevant regime has been missing from our ability to quantitatively measure and thus understand deformation in glasses: the low temperature and high strain rate regime, i.e. deformation conditions which correspond to the drop of a mobile device or the exposure of windows to sand-rich winds. Here, we demonstrate for the first time how these conditions may be studied quantitatively and systematically and that four distinctly different glasses show the same behaviour towards the extreme conditions at high rates and low temperatures far below the glass transition temperature. The presented insights enable an easy comparison between different materials and a preselection without having to conduct extensive experiments with different materials, simplifying the design process considerably.

The practical use of glasses is strongly linked to the resistance against crack initiation and more importantly crack growth, i.e. the toughness of a glass. These properties in turn are also determined by the dominant deformation mechanisms of flow. Depending on whether the glass deforms mainly by volume conservative shear or densification – compaction of the glass structure - it is referred to as normal or anomalous, respectively^1,2^. Activation of either results in different cracking behaviour^3^ and depends not only on the structure and chemistry of the glass but also on the environmental and loading conditions, e.g. temperature and strain rate. The response of glasses to high strain rates is of special interest as a change in deformation characteristics has been seen with increasing strain rate for numerous glasses at high temperatures: at low strain rates, their viscosity is constant resulting in a linear increase of stress with increasing strain rate - Newtonian behaviour^4^. With increasing strain rate, deformation enters a non-Newtonian regime, where the viscosity decreases and the rate at which the stress increases with strain rate therefore decreases and finally converges to a constant level, becoming independent of the strain rate^4,5^. Whether this behaviour extends to low temperatures in glasses was previously unknown.

Up to now, it has simply not been possible to obtain detailed information from deformation experiments conducted both at room temperature and at high strain rates and allowing a quantitative analysis of deformation modes, e.g. activation of shear, densification and critical stress levels. (Nano)indentation, micropillar compression and diamond anvil cell experiments can be used to introduce plastic deformation at temperatures far below the glass transition temperature T_g_, however, these are all normally limited to low strain rates^6,7^. At high temperature, fibre pulling and conventional macroscopic compression experiments^8^ can be used to apply high strain rates, but these methods cannot be used at low temperatures as they result in premature fracture from pre-existing flaws^9^. Impact testing allows studies at high rates and variable temperature. However, classical methods, even in combination with high speed imaging, are still limited in terms of their quantitative interpretation and, due to the projectile size, the typical sample volumes within bulk samples are large enough to also contain statistically distributed intrinsic flaws governing deformation^10^.

**Figure 1:**
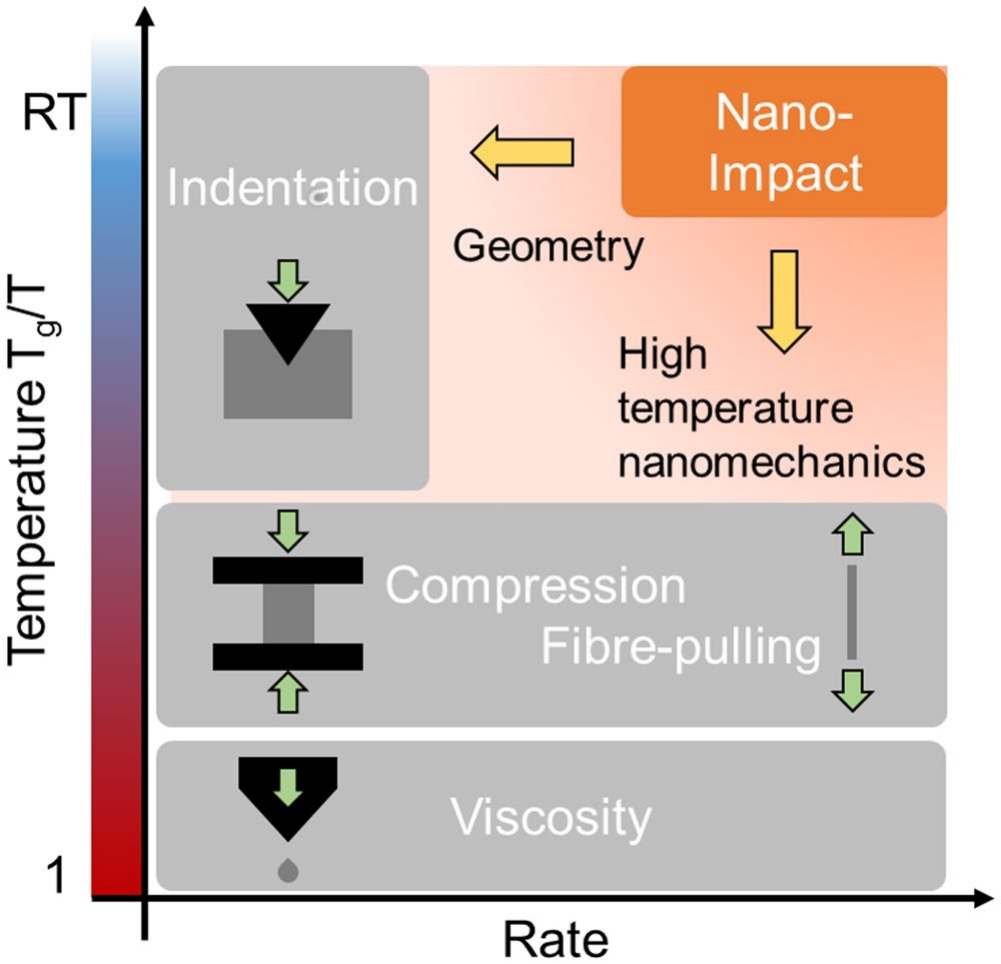
Previously inaccessible low temperature and high rate deformation regime now covered by impact nanoindentation testing. The experimental method in combination with in-depth analysis is envisioned to bridge the present characterisation gap between already available high temperature and indentation methods. Development of the required equipment has already been achieved in the context of other nano-mechanical test methods^11–13^.

In contrast, nano-impact testing, i.e. instrumented (nano) indentation in a ballistic, high rate operation, offers the possibility to deform glasses plastically to high rates at room temperature. This is achieved by employing the same geometry as in quasi-static indentation and therefore inducing a confining pressure that suppresses fracture and enables plastic deformation in indentation of brittle materials in general. Until now, this approach only has found limited use in the characterisation of fused silica^11^, sol-gel coatings^12^ and magnesium alloys^13^. Its application is often limited by the oscillations that develop in an un-stiffened pendulum. Using the stiffening modifications shown to be effective by Jennet *et al*.^11^, impact nanoindentation is now utilised here and applied to very high strain rates beyond the scope of conventional hardness measurements. The latter is realized by accelerating the tip into the sample by releasing a controlled elastic deformation introduced into the tip mount, and collecting the position-time-signal at a high acquisition rate during impact and several subsequent rebounds. By using different tip geometries and a high temperature setup, such experiments could span a wide range of strain rates (10^−4^–10^5^ s^−1^) and temperatures (≈150–1200 K)^14,15^ entering regimes which have not been accessible so far by conventional methods (Fig. 1).

## Results and Discussion

In this study, different glass systems have been tested at room temperature over strain rates spanning seven orders of magnitude by using quasi-static nano-indentation and dynamic impact-nanoindentation. To cover a maximum diversity of glasses, the most anomalous glass, fused silica, and a glass presenting the normal, shear dominated extreme, a bulk metallic glass (BMG), were chosen. Additionally, a sodium-borosilicate glass (NBS) was subjected to two different thermal treatments resulting in an open structure (quenched) and a dense structure (annealed) and included into this study to change the extent of normal and anomalous behaviour within one glass composition^16,17^. To vary the hydrostatic stress component and hence the relative fraction of densification during deformation^18^, the NBS glasses were additionally tested with two different tip geometries: a sharp cube corner and a comparatively flat Berkovich tip.

While it is well known how these glasses behave at low strain rates and room temperature^6,19–21^, there is no information how shear and densification, stress and viscosity develop towards high rates at room temperature. To obtain this crucial information, a new methodology is presented here analysing different parameters of the position-time-data recorded in each experiment during impact nanoindentation. Two of these are of special interest: the energy loss ratio and the dynamic hardness. The energy loss ratio represents the energy dissipated during impact and thus the ratio of plastic and elastic deformation; an increasing energy loss ratio corresponds to a shift from elastic to plastic deformation. The dynamic hardness gives a measure of the resistance to impact deformation by taking the dissipated energy over the volume plastically displaced by the tip during the very first impact.

**Figure 2:**
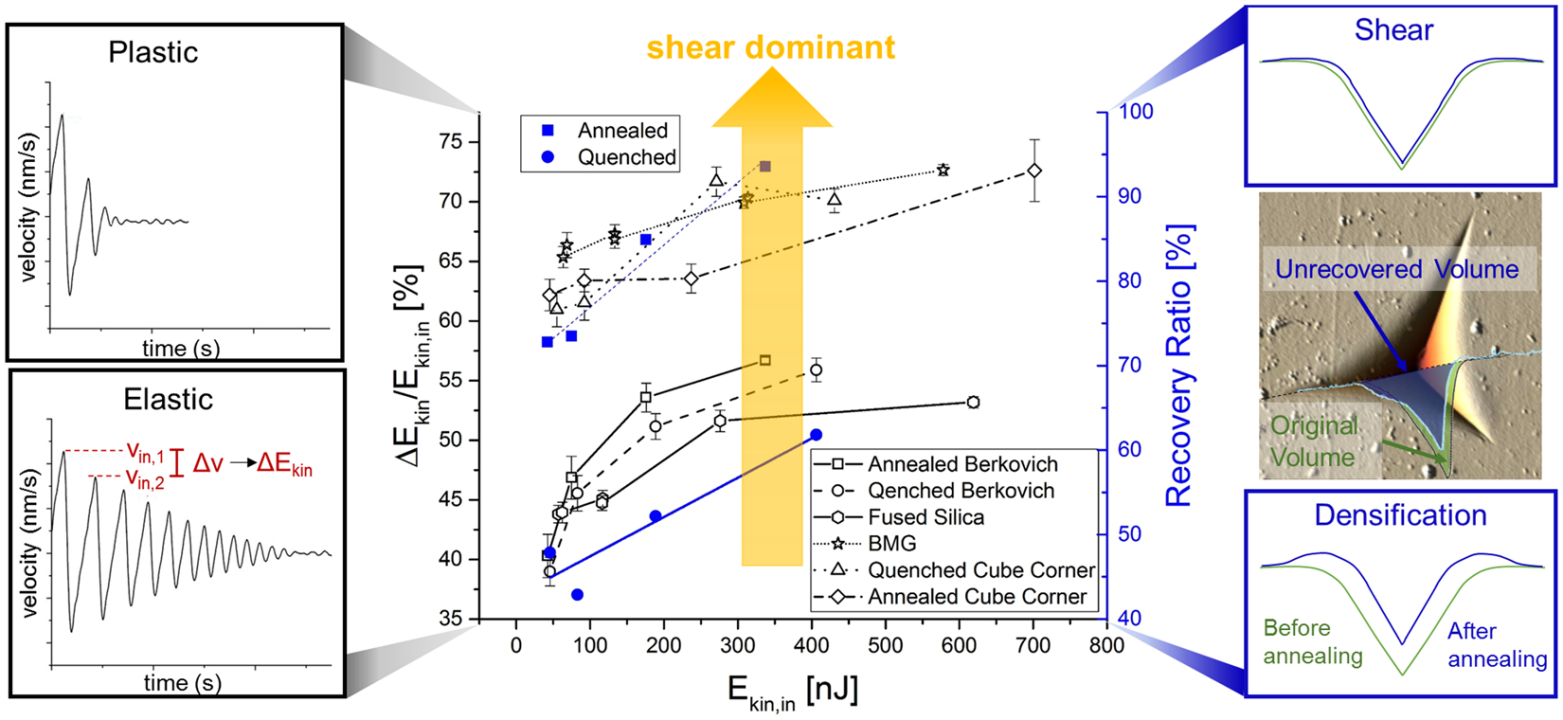
Influence of strain rate (determined by the plotted kinetic impact energy) on the ratio of shear and densification as well as plastic and elastic deformation. The energy loss ratio from the velocity-time data of impact-nanoindentation for different glasses yields consistent behaviour with the recovery ratio for the indented volume derived from annealing experiments and subsequent AFM analysis. An increasing amount of shear deformation is correlated with an increase in energy loss, or plastic deformation, during the first indent and found, consistent with glass structure and stress state, towards the densest glass networks and smaller hydrostatic stress component.

In terms of the energy loss ratio, all samples show the same trend (Fig. 2): an increase of energy loss ratio and thus plastic deformation with increasing strain rate (determined by the impact energy). AFM measurements show that the samples tested with the Berkovich tip do not form cracks so that the complete energy loss is due to plastic deformation and not influenced by cracking. To investigate the amount of densification and shear contributing to the plastic deformation at increasing rates, annealing treatments were conducted on the two NBS glasses after impact indentation. It was found that both samples reveal a shift from densification to shear deformation with increasing strain rate, so that the increase in plastic deformation - indicated by the increase in energy loss ratio - appears to be accommodated to a greater extend by shear flow as the strain rate increases. Interestingly, the related changes in energy loss ratio are consistent with this transition both in terms of the dominant deformation mechanisms for each glass and the induced stress components: an increased shear flow component is expected in glasses where densification is less dominant and where the impact tip geometry is sharper, reducing the hydrostatic pressure. This is reflected in the experiment in terms of the energy loss ratio enveloped by the open fused silica structure at the lower and the dense bulk metallic glass at the upper end as well as an increase in energy loss ratio from the flat Berkovich geometry to the sharper cube corner tip.

These data suggest that an increasing strain rate shifts the governing mechanism of deformation towards shear flow in the investigated glasses showing both densification and shear. We then measured the hardness and viscosity across seven orders of magnitude. At high temperatures, measurements of the viscosity reported in the literature have identified non-Newtonian flow^22^. A central question is therefore how this non-Newtonian flow might change, or not, as the temperature is reduced to room temperature, i.e. the brittle regime where macroscopically deformation is governed by fast fracture from statistically distributed flaws.

**Figure 3:**
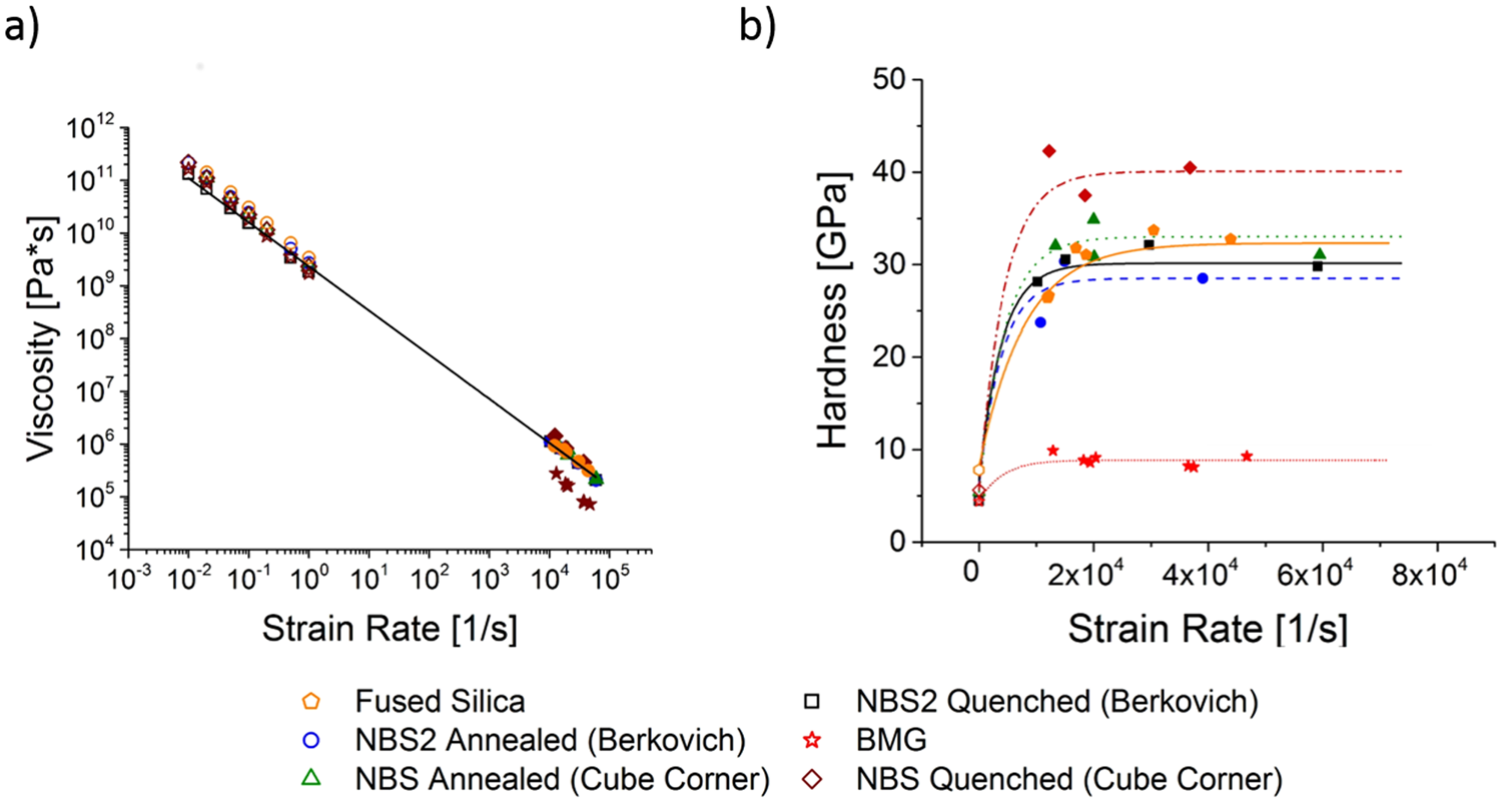
Dependence of (**a**) viscosity and (**b**) hardness on increasing strain rate for different materials tested by quasi-static and impact nanoindentation across seven orders of magnitude. The viscosity exhibits a linear change with strain rate in double logarithmic presentation (see Fig. 4 for a double logarithmic plot of the normalised hardness). The filled symbols correspond to the impact indentation tests, the empty ones to quasi-static indentation.

Non-Newtonian behaviour manifests in two characteristic trends: a decreasing viscosity and therefore a decreasing rate of stress increase with strain rate, after which the stress converges to a constant value at high strain rates. Over the complete range of measurements, the viscosity decreases linearly (Fig. 3), connecting the quasi-static and the impact measurements across seven orders of magnitude from indentation/impact strain rates of ${10}^{-2}{{\rm{s}}}^{-1}$ to ${10}^{4}{{\rm{s}}}^{-1}$. This linear drop in viscosity is in excellent agreement with the expected change in viscosity for a typical shear thinning material, as has been shown for glasses near T_g_ but has not been possible to show directly in experiments at room temperature before^4,5,23–25^.

Parallel to this linear drop in viscosity, the hardness rises towards higher rates and at the highest rates, converges towards a single value or, in a linear representation, exhibits a plateau stress (Fig. 3b). Together with the decreasing viscosity, the transition between these two deformation regimes demonstrates for the first time the existence of non-Newtonian behaviour with shear thinning at room temperature.

In absolute terms, the limit stresses for each of the different glasses varies, just as the other physical parameters describing structure, bond strength or density. Most commonly, the limit strength at high rates and temperatures has been associated with structural rearrangements and it is assumed that the stress level is determined by the theoretical strength, *σ*_*th*_, which can be derived as^5^:

1 $${\sigma }_{th}=\sqrt{\frac{E\gamma }{{r}_{o}}}$$

with E the elastic modulus, γ the interface energy and r_o_ the atomic equilibrium distance.

**Figure 4:**
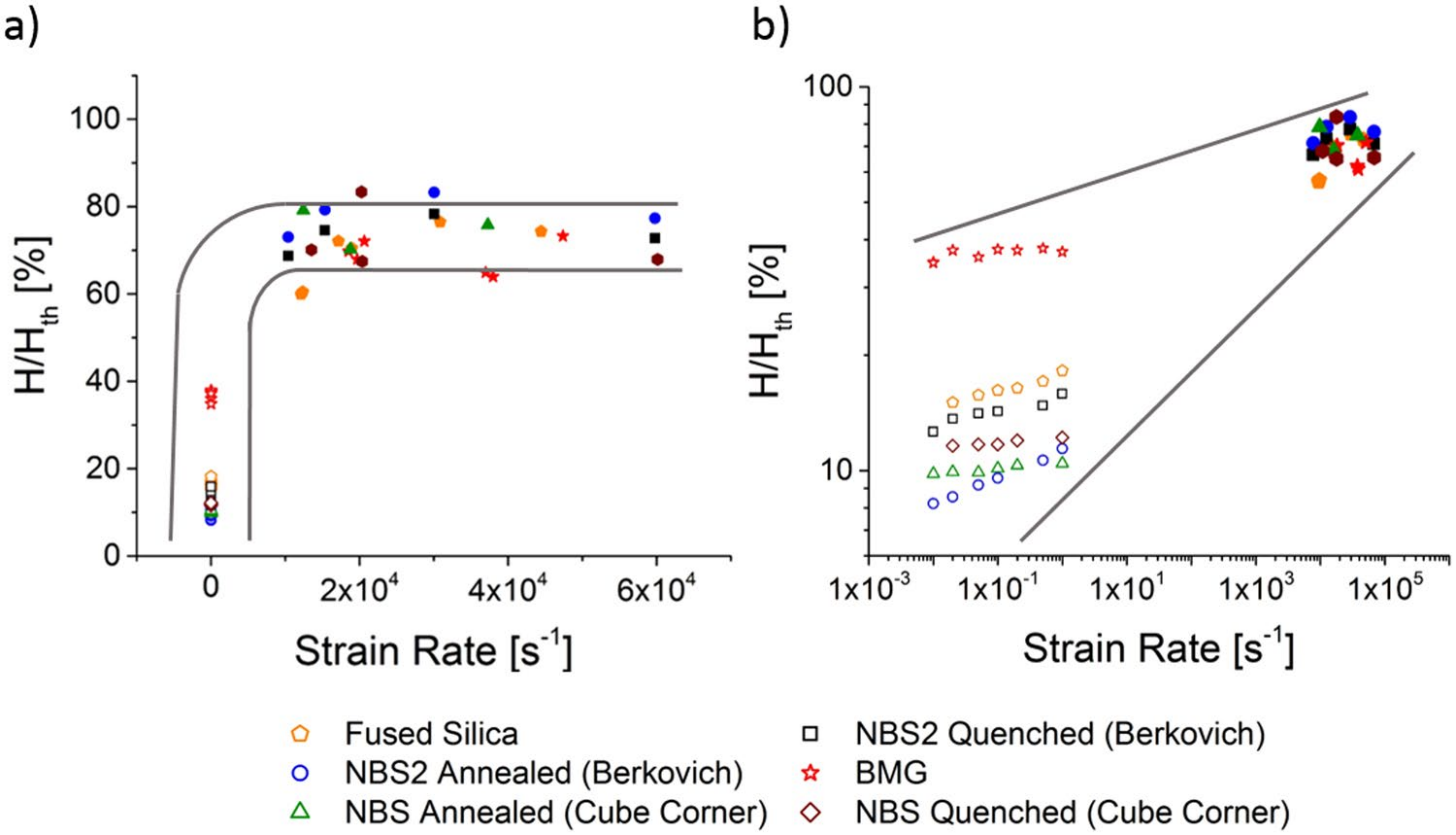
Hardness during quasi-static and impact nanoindentation of different materials normalized by their theoretical hardness over indentation/impact strain rate. The normalized hardness converges to approximately 75% H_th_ at high rates for all glasses. The high and low rate regimes are expanded for clarity in an (**a**) linear and (**b**) logarithmic scaling of the rate axis, respectively.

Taking the theoretical strength and modulus, the theoretical hardness for every material tested was derived. According to Marsh^26^, the glass strength can be converted into a hardness using a constraint factor, which depends on the material and in particular the relative importance of plastic and elastic deformation, i.e. the ratio of strength to elastic modulus (values used for each glass are given in the Materials and Methods section). All four glasses show a plateau at approximately 75% of their theoretical hardness (Fig. 4). This implies not only that non-Newtonian flow takes place in all samples at room temperature but also that the way it develops is similar in all of them. This result is of particular interest as the samples chosen span the complete range of deformation modes in glasses starting at the anomalous extreme in fused silica over two boro-silicate glasses showing both densification and shear in different amount to the normal, shear dominated extreme of a BMG.

## Conclusions

Despite a manifold of experimental and theoretical evidence near the glass transition, it has not been possible to extract the exact mechanisms governing non-Newtonian flow and the importance of the theoretical strength in glasses. The extension of the experiments to a temperature far below T_g_ will therefore support the quest for the atomistic mechanisms of deformation at high rates by legitimizing interpretation of atomistic simulations at small scales and at short time scales via the now available experiments and additional insights at room temperature. These not only give evidence to continuous flow mechanisms across temperatures at high rates, where thermal activation becomes limited, but also provide an essential approach to guide modelling of glass deformation. In extending the large available datasets and models for mechanical properties at low rates, it is now possible to derive an approximate strength at high strain rates in a simple way from basic glass properties by deriving the theoretical strength.

The approach and insights presented here, employing impact nanoindentation to reveal shear thinning in different glasses at room temperature, therefore offer the possibility to choose glasses and characterize or model their performance in the highly relevant room temperature impact regime more efficiently. In particular, an easily applied method to characterize flow at high rates and low temperature is now available to researchers, as well as for developers facing a choice of glass system for devices or components exposed to impact loading. It has been demonstrated that modelling of high rate deformation may perhaps simply be approximated by non-Newtonian flow limited by the theoretical strength across glass structures.

## Materials and Methods

Different glass samples were investigated: fused silica, a bulk metallic glass (BMG) and two sodium-borosilicate (NBS) samples of the same glass, but subjected to different thermal treatments. The NBS glass (74SiO_2_–20.7B_2_O_3_-4.3Na_2_O-1Al_2_O_3_) was melted and homogenized at 1650 °C. The melt was then cast either onto a brass block (left at room-temperature) or into a graphite mould, which had been preheated to 700 °C. The 700 °C mould was furthermore slowly cooled down to room temperature with 30 K/h, resulting in a relaxed, compact structure, this state is henceforth referred to as “annealed”^16,17^. The sample cast onto the brass block was additionally quenched by another brass stamp to quickly dissipate the heat, resulting in an open structure, and is henceforth referred to as “quenched”^27^. The BMG used in this study was a ‘Vitreloy‘ from Liquid metals, a Zr_41.2_Be_22.5_Ti_13.8_Cu_12.5_Ni_10.0_ metallic glass.

For quasi-static indentation (iNano, Nanomechanics Inc., USA), a maximum load of 45 mN was chosen and strain rates ranging from 0.01 to 1 s^−1^ were applied using a diamond Berkovich tip.

AFM measurements (XE-70¸Park Systems) were conducted with scan speeds of 0.1 µm/s and a resolution of 512 × 512 pixels.

**Figure 5:**
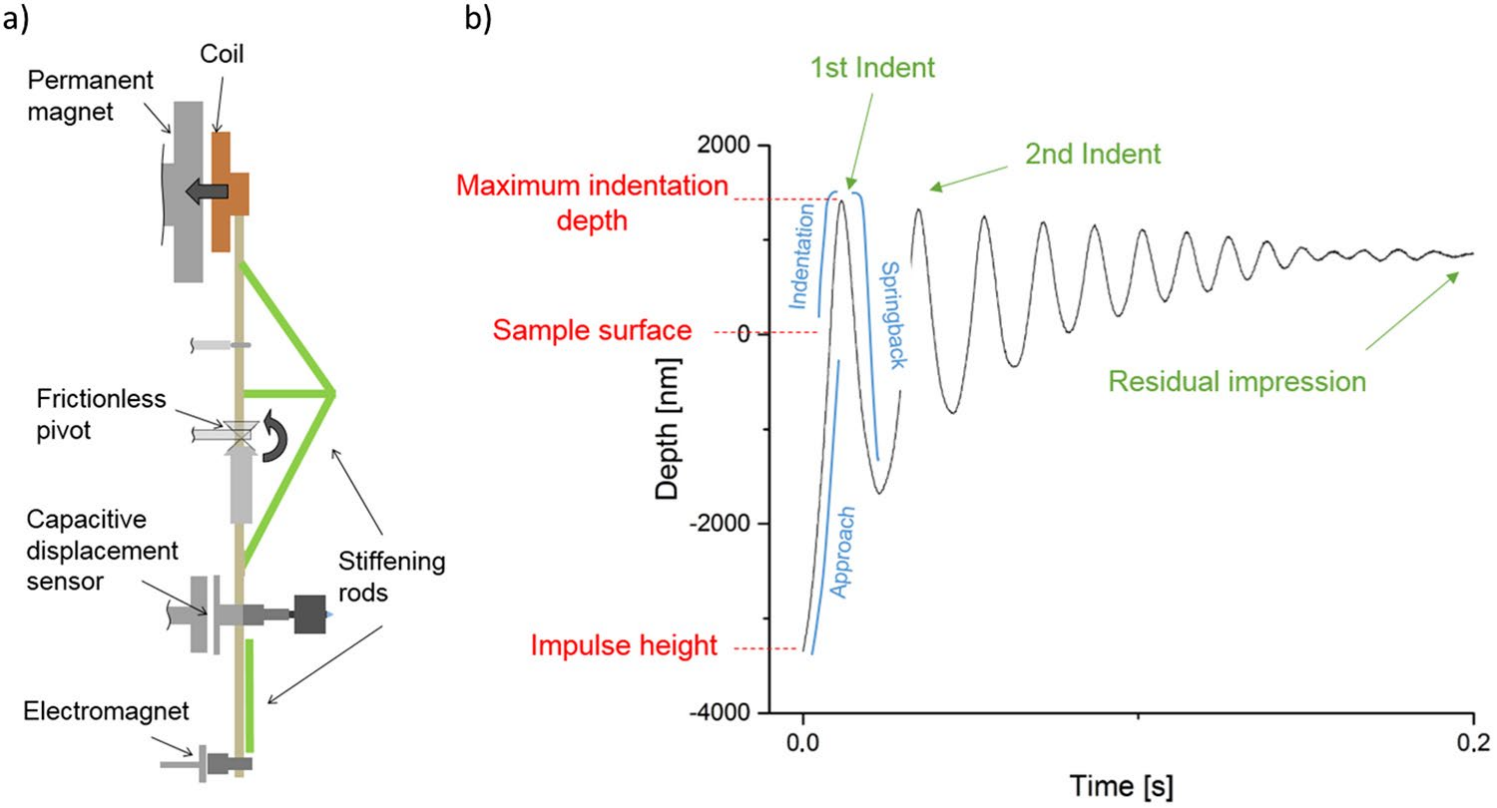
Experimental setup of the impact nano-indenter (**a**). By applying voltage to the coil on the top end of the pendulum, a rotation around the pivot will be induced, resulting in a forward movement of the tip. The lower electromagnet can be used to stress the pendulum and accelerate it quickly towards the sample. A typical depth-time curve (**b**) is displayed with the important parameters marked in the graphs.

For impact nanoindentation, a pendulum-based system was used (Fig. 5a) (NanoTest Platform III, Micromaterials Ltd.). By applying a voltage to the coil on the top end of the pendulum, a rotation around the pivot is induced, resulting in a forward movement of the tip. Prior to this, the lower electromagnet can be used to stress the pendulum. By releasing it, the pendulum swings forward with a high acceleration, resulting in a nano-impact indentation on the sample. These impact nanoindentation experiments were conducted using loads between 10 mN and 100 mN with an initial distance between tip and sample in the stressed state of 5 µm. At every load 15 tests were conducted per sample. A flat Berkovich tip and a sharp cube corner tip were used, both consisted of diamond.

In impact nanoindentation, the tip hits the sample with a high velocity, indenting by producing elastic and plastic deformation. At the point of maximum indentation depth, the elastic deformation will be reversed, resulting in a backward movement of the tip. This will even cause the tip to leave the sample and be stressed again. This procedure repeats several times until all energy is absorbed by the sample. Figure 5b displays a typical depth-time curve. Although this results in repeated impacts of the sample, only the first impact is considered in the analysis of the materials: dynamic hardness, energy loss ratio and strain rate (detailed subsequently) are all calculated from the initial contact.

Several important parameters can be determined from these data: from the depth-time curve, velocity-time curves can be derived. By using an effective mass of the pendulum, the kinetic energy can be estimated. With this information the dynamic hardness and the energy loss ratio are calculated. To derive the dynamic hardness, H_dyn_, the energy loss between the first and the second indent is taken, derived from the maximum velocities right before the indenter hits the sample for the first and the second time^12,28^. Here improved data quality allows us to use the plastic volume of the first impact, *V*_*plast*_, rather than the conventionally used volume of the final impression, giving:

2 $${H}_{dyn}=\frac{\frac{1}{2}{m}_{eff}({v}_{in,2}^{2}-{v}_{in,1}^{2})}{{V}_{plast}}$$

with m_eff_ the effective mass of the pendulum, v_in,1_ and v_in,2_ the maximum velocities right before the indent hits the sample for the first and the second time, respectively,  and V_plast_ the plastic volume after the first impact.

The second important parameter is the energy loss ratio (ELR):

3 $$ELR=\frac{{E}_{kin,2}-{E}_{kin,1}}{{E}_{kin,1}}$$

with E_kin,1_ and E_kin,2_ being the kinetic energy of the pendulum right before the first and second indent, respectively. This value describes the amount of energy lost during the first impact, i.e. the amount of plastic deformation. By normalising it to the kinetic energy of the pendulum before the first impact, the ratio between elastic and plastic deformation can be analysed with a high ELR corresponding to a high amount of plastic deformation. It can be shown that the maximum kinetic energy right after leaving the sample and hitting it the next time is the same so that it can be stated that no energy is lost during the springback of the pendulum between two indentations. Also the ELR shows similar trends for the samples tested with a Berkovich and a cube corner tip. Interestingly, the Berkovich indents do not show cracking while the cube corner indents all show cracking. This means that the effect of cracking on the ELR is not dominant.

To analyse the data in a physically meaningful way, the strain rate must be determined. For this, the typical indentation strain rate^29^ was used:

4 $$\dot{\varepsilon }=\frac{\dot{h}}{h}$$

with h being the indentation depth and $\dot{h}$ being the velocity. As the strain and stress field during indentation is spatially inhomogeneous, the strain rate, hardness, viscosity or even the modulus have to be considered as a representative value for a certain volume (and direction in anisotropic materials), in analogy to the hardness being interpreted as the flow stress at a specific strain of 8%^30^. This makes direct comparison of indentation test to uniaxial measurements challenging. Nevertheless, it has been shown that the indentation strain rate determined by equation 4 can be set to be constant during an indentation experiment and under these conditions good correlation with uniaxial testing is commonly observed^29,31–33^.

Different to quasi-static indentation, where the strain rate can be controlled throughout the complete experiment, the strain rate decreases constantly with increasing indentation depth in impact nano-indentation. To compare different measurements, one depth must be selected at which the strain rate is measured as the representative strain rate of the experiment. As tip rounding will have a very strong influence on the measured values below 50 nm, especially in impact mode, in this study the strain rate at 50 nm was taken as the strain rate of the complete indentation cycle to compare different experiments. With these dynamic hardness and strain rate values, the viscosity can be determined in indentation experiments by the following equation^34^:

5 $$\eta =\frac{{H}_{dyn}}{2(1+\nu )\dot{\varepsilon }}$$

with η the viscosity and *v* the Poisson ratio.

In order to investigate the amount of shear and densification, AFM measurements were conducted. When glasses are subjected to temperatures near the glass transition, here 0.9 T_g_ for 2 hours, it has been shown that densification can be fully reversed^18^ while shear deformation is still overwhelmingly present. By measuring the volume using AFM before and after annealing, the amount of unrecovered volume can be determined and in this way a recovery ratio, the unrecovered volume divided by the initial volume, can be derived constituting the amount of unrecovered deformation, produced by shear that is largely — if not entirely — unaffected by a short annealing treatment^18^.

In order to compare the different materials, the theoretical strength was calculated and transferred into a theoretical hardness using a constraint factor according to Marsh^26^. Table 1 lists the parameters for theoretical strength, constraint factor and hardness used for the different materials:

**Table 1:** Theoretical Strength, constraint factor and theoretical hardness for the different materials tested.

Material	Theoretical strength [GPa]	Constraint factor	Theoretical hardness [GPa]
BMG	5.46	2.33	12.73
Fused Silica	35.54	1.25	44.50
NBS Quenched (Berkovich)	30.45	1.36	41.37
NBS Annealed (Berkovich)	30.45	1.28	38.89
NBS Quenched (cube corner)	30.45	1.52	46.17
NBS Annealed (cube corner)	30.45	1.77	53.92

## References

[1] Arora A, Marshall DB, Lawn BR, Swain MV (1979). Indentation deformation/fracture of normal and anomalous glasses. Journal of Non-Crystalline Solids.

[2] Peter KW (1970). Densification and flow phenomena of glass in indentation experiments. Journal of Non-Crystalline Solids.

[3] Cook RF, Pharr GM (1990). Direct Observation and Analysis of Indentation Cracking in Glasses and Ceramics. Journal of the American Ceramic Society.

[4] Yue Y, Brückner R (1994). A new description and interpretation of the flow behaviour of glass forming melts. Journal of Non-Crystalline Solids.

[5] Simmons JH, Mohr RK, Montrose CJ (1982). Non‐Newtonian viscous flow in glass. Journal of Applied Physics.

[6] Schuh CA, Nieh TG (2003). A nanoindentation study of serrated flow in bulk metallic glasses. Acta Materialia.

[7] Dubach A, Raghavan R, Löffler JF, Michler J, Ramamurty U (2009). Micropillar compression studies on a bulk metallic glass in different structural states. Scripta Materialia.

[8] Harmon JS, Demetriou MD, Johnson WL, Samwer K (2007). Anelastic to Plastic Transition in Metallic Glass-Forming Liquids. Physical Review Letters.

[9] Griffith AA (1921). The Phenomena of Rupture and Flow in Solids. Philos. Trans. R. Soc. London, Ser. A.

[10] Chaudhri MM, Liangyi C (1986). The catastrophic failure of thermally tempered glass caused by small-particle impact. Nature.

[11] Jennett M, Nunn J (2010). High resolution measurement of dynamic (nano) indentation impact energy: a step towards the determination of indentation fracture resistance. Philosophical Magazine.

[12] Wheeler JM, Gunner AG (2013). Analysis of failure modes under nano-impact fatigue of coatings via high-speed sampling. Surface and Coatings Technology.

[13] Somekawa H, Schuh CA (2012). High-strain-rate nanoindentation behavior of fine-grained magnesium alloys. Journal of Materials Research.

[14] Harris AJ, Beake BD, Armstrong DEJ (2015). Extreme nanomechanics: vacuum nanoindentation and nanotribology to 950 °C. Tribology - Materials, Surfaces & Interfaces.

[15] Lee S-W, Meza L, Greer JR (2013). Cryogenic nanoindentation size effect in [0 0 1]-oriented face-centered cubic and body-centered cubic single crystals. Applied Physics Letters.

[16] Zehnder, C. *et al*. Influence of Cooling Rate on Cracking and Plastic Deformation during Impact and Indentation of Borosilicate Glasses. *Frontiers in Materials* **4**, 10.3389/fmats.2017.00005 (2017).

[17] Malchow P (2015). Composition and cooling-rate dependence of plastic deformation, densification, and cracking in sodium borosilicate glasses during pyramidal indentation. Journal of Non-Crystalline Solids.

[18] Yoshida S, Sanglebœuf J-C, Rouxel T (2005). Quantitative evaluation of indentation-induced densification in glass. Journal of Materials Research.

[19] Schuh CA, Hufnagel TC, Ramamurty U (2007). Mechanical behavior of amorphous alloys. Acta Materialia.

[20] Demetriou, M. D. *et al*. A damage-tolerant glass. *Nature Materials* **10**, 123–128, http://www.nature.com/nmat/journal/v10/n2/abs/nmat2930.html#supplementary-information (2011). 10.1038/nmat2930 21217693

[21] Kermouche G, Guillonneau G, Michler J, Teisseire J, Barthel E (2016). Perfectly plastic flow in silica glass. Acta Materialia.

[22] Simmons JH (1998). What is so exciting about non-linear viscous flow in glass, molecular dynamics simulations of brittle fracture and semiconductor–glass quantum composites. Journal of Non-Crystalline Solids.

[23] Simmons JH, Ochoa R, Simmons KD, Mills JJ (1988). Non-Newtonian viscous flow in soda-lime-silica glass at forming and annealing temperatures. Journal of Non-Crystalline Solids.

[24] Kato H, Kawamura Y, Inoue A (1998). Newtonian to non-Newtonian master flow curves of a bulk glass alloy Pd40Ni10Cu30P20. Applied Physics Letters.

[25] Brückner, R., Yue, Y. & Habeck, A. Determination of rheological properties of high-viscous glass melts by the cylinder compression method. *Glass Science and Technology* **67** (1994).

[26] Marsh DM (1964). Plastic Flow inGlass. Proc. R. Soc. London, Ser. A.

[27] Doris Moncke, Tricot G, Doris Ehrt, Efstratios I, Kamitsos (2015). Connectivity of Borate and Silicate Groups in a low-alkali Borosilicate Glass by Vibrational and 2D NMR Spectroscopy. Journal of Chemical Technology and Metallurgy.

[28] Schuh JRTCA (2008). The Hall–Petch breakdown at high strain rates: Optimizing nanocrystalline grain size for impact applications. Applied Physics Letters.

[29] Lucas, B. N. & Oliver, W. C. Indentation power-law creep of high-purity indium. *Metallurgical and Materials Transactions**A* **30**, 601–610 (1999).

[30] Tabor D (1970). The hardness of solids. Review of Physics in Technology.

[31] Maier V (2011). Nanoindentation strain-rate jump tests for determining the local strain-rate sensitivity in nanocrystalline Ni and ultrafine-grained Al. Journal of Materials Research.

[32] Schwaiger R, Moser B, Dao M, Chollacoop N, Suresh S (2003). Some critical experiments on the strain-rate sensitivity of nanocrystalline nickel. Acta Materialia.

[33] Maier-Kiener V, Durst K (2017). Advanced Nanoindentation Testing for Studying Strain-Rate Sensitivity and Activation Volume. JOM.

[34] Guin J-P (2002). Indentation creep of Ge–Se chalcogenide glasses below Tg: elastic recovery and non-Newtonian flow. Journal of Non-Crystalline Solids.

